# Artificial insemination with fresh, liquid stored and frozen thawed semen in dromedary camels

**DOI:** 10.1371/journal.pone.0224992

**Published:** 2019-11-07

**Authors:** Samir Al-Bulushi, Bodhaganahalli M. Manjunatha, Roslyn Bathgate, Jessica P. Rickard, Simon P. de Graaf

**Affiliations:** 1 The University of Sydney, Faculty of Science, School of Life and Environmental Sciences, NSW, Australia; 2 Animal Research Centre, Directorate General of Veterinary Services, Royal Court Affairs, Muscat, Oman; 3 The University of Sydney, Faculty of Science, Sydney School of Veterinary Science, NSW, Australia; University of Florida, UNITED STATES

## Abstract

This study was conducted to evaluate various factors affecting fertility following insemination of dromedary camels. In experiment 1, camels were either bred by natural mating (NM) or inseminated in the body of uterus with whole, split (50:50) or 1 mL of undiluted ejaculate. In experiment 2, camels were inseminated with fresh diluted semen either in the body of the uterus or tip of the uterine horn and at either the time of ovulation induction (0 h), 24 or 30 h later. In experiment 3, camels were inseminated at the tip of the uterine horn with different doses of fresh diluted semen (75, 150 or 300 x 10^6^ motile spermatozoa) or with 150 x 10^6^ motile spermatozoa diluted with different extenders (Green buffer, Optixcell or Triladyl). In experiment 4, camels were inseminated in the tip of the uterine horn with diluted (Triladyl or Optixcell) liquid-stored semen or diluted (Triladyl) frozen-thawed semen consisting of either 300 or 500 x 10^6^ motile spermatozoa. The pregnancy rate in camels bred by NM was similar to camels inseminated with whole undiluted ejaculates whereas insemination with 1 mL undiluted ejaculate resulted in lower pregnancy compared to whole and split undiluted ejaculates (*P < 0*.*05*). Deposition of semen in the uterine body resulted in lower pregnancy rates compared to deposition in the tip of the horn (35.3% *versus* 72.2%, *P < 0*.*05*) but insemination at the time of ovulation induction and 24 h later resulted in higher pregnancy rate to camels inseminated at 30 h after induction (68.4 and 70.0% versus 23.5%; *P < 0*.*05*). Artificial insemination with 75 x 10^6^ motile spermatozoa resulted in lower pregnancy rates compared to 150 and 300 x 10^6^ motile spermatozoa doses (40.9% *versus* 65.2 and 70.0%, respectively) and pregnancy rate was not affected by extenders. Insemination of chilled motile spermatozoa stored in either Triladyl or Optixcell resulted in similar pregnancy rates, regardless of insemination dose, although an upward trend with increasing sperm number was apparent (Triladyl; 11.1% *versus* 21.1% and Optixcell; 5.9% versus 12.5%, for 300 x 10^6^ and 500 x 10^6^ groups, respectively; *P > 0*.*05*). No pregnancies were obtained with frozen thawed semen. In conclusion, this study demonstrated that the success of camel AI is highly dependent on sperm dose, location of semen deposition, timing of insemination and semen type. Further studies are required to determine the reason for the compromised fertility of preserved semen despite apparent high *in vitro* quality.

## Introduction

Artificial insemination (AI) is used for the rapid dissemination of elite genetic material over large geographic areas in several domestic animals. Over the last two decades, there has been increased interest in the breeding of genetically superior camels using embryo transfer and AI technologies, due to the growing commercial importance of camels in the Gulf countries of the Middle Eastern region. Although the first camelid offspring from AI was reported in Bactrian camels in 1961 [1], AI protocols are yet to be established in dromedary camels to the same extent as they are in the major domestic animal species. This is due to a variety of difficulties including complications associated with semen collection, bull sexual behavior, the quality of semen produced (low volume, low sperm concentration and high viscosity), limited knowledge of optimum insemination time and sperm dose and lack of a standard storage technique [2, 3].

At present, bulls of proven fertility are housed individually under an intensive management system and used for hand mating across Oman in several Government and privately owned camel farms. Several studies have been performed on semen collection and storage methods in camels [3–9]. To date, some success has been achieved using fresh semen for AI in this species. Artificial insemination with raw semen resulted in a 40–46.1% pregnancy rate [4, 10]. However, the results (pregnancy rates) were lower compared to timed natural mating programs (67–71.4%) [11,12]. Pregnancy rates have been reported after AI of fresh semen diluted in lactose plus egg yolk (50%, [13]), Laciphos (53%, [14]), skimmed milk (0%, [14]), Green buffer plus egg yolk (10–72.7%, [8, 15, 16]) and INRA 96 (34%, [8]). Although many aspects of factors affecting the success of AI have not been studied, some researchers have studied the effect of site of insemination [15], number of spermatozoa inseminated [8, 15] and timing of insemination in relation with ovulation [17, 18] on pregnancy rate. The use of chilled semen for AI resulted in low pregnancy rates (0–37.5%) [8, 10]. Although, one group reported a 95% pregnancy rate after AI with frozen-thawed semen [19] later studies have reported only 7.1% pregnancy [4] and a recent study reported live birth of calves following frozen thawed semen insemination [20], but with a paucity of detail with respect to the methods employed to achieve such an outcome.

To facilitate the successful implementation of AI in camels, the optimum semen extenders, insemination dose, timing and site of insemination must all be established. The present study aimed to investigate the effect of these factors on success of AI in dromedary camels in an effort to improve pregnancy rates not only with fresh semen but chilled and frozen semen as well.

## Materials and methods

These experiments were approved by The Animal Welfare Committee of Animal Health Research Centre, Directorate General of Agriculture and Livestock Research, Ministry of Agriculture and Fisheries, Oman.

### Semen extenders

Composition of the extenders used, as provided by the suppliers are: Green buffer (IMV Technologies, France) is an egg yolk-based extender and the composition is undisclosed. Optixcell (IMV Technologies, France) is a chemically-defined, animal protein-free extender, which contains liposomes, carbohydrate, mineral salts, buffer, antioxidants, glycerol, tylosin, gentamicin, spectinomycin and lincomycin. Triladyl (Minitub, Tiefenbach, Germany) is egg yolk-based extender which contains tris, citric acid, fructose, glycerol, tylosin, gentamicin, spectinomycin and lincomycin. The preparation of each extender was carried out according to the manufacturer instructions. Extenders were prepared fresh on the morning of each semen collection day and total volumes of extenders were prepared based on the anticipated volume of semen to be processed. For egg yolk-based extenders (Triladyl and Green buffer), 20% egg yolk was mixed at room temperature on the day of semen collection, as per manufacturer instruction.

### Animals

This study was conducted at the Animal Research Centre, Royal Camel Corps, Muscat, Sultanate of Oman, during the peak breeding season of November to March (2015–2018). Adult dromedary male camels aged between 8–14 years, with a history of normal fertility (≥ 60% conception) in natural breeding programs were selected for this study and were housed in individual pens. Six dromedary male camels were used exclusively for semen collection and three male camels were used for natural mating. Female adult camels with normal reproductive tracts, as per-vaginal and transrectal ultrasonographic examinations, body condition score of 3.4 ± 0.4 (Scale 1–5, 1 = emaciated and 5 = obese) were selected. These camels were housed in pens isolated from the males and had free access to water. Animals were fed fresh green grass/dry fodder, as well as commercial concentrate pellets supplemented with minerals. In this study, camels that were diagnosed as pregnant after breeding were moved to separate paddocks to manage pregnancy. Camels that failed to conceive or underwent early pregnancy loss were bred by natural service or used as recipients in embryo transfer programmes or used in other research projects.

### Semen collection

Semen was collected at weekly intervals as described previously [21] using a bovine artificial vagina (AV; 30 cm length and 5 cm internal diameter). A smooth latex liner was mounted and fixed at both ends of the AV and a transparent graduated glass water-jacketed semen collection vessel was attached to the apex of the internal latex liner. The inner chamber of the AV was filled with water (45–48°C) in order to maintain an internal AV temperature of 41–42°C during semen collection. The water-jacketed semen collection vessel was then filled with warm water (37°C) and the inner surface of the AV was lubricated with a sterile K-Y lubricating jelly (Johnson and Johnson, Sezanne, France). Prior to semen collection, bulls were exposed to a sexually receptive female for about a period of 10 min, after which the bull was allowed to approach and mount a female camel restrained in sternal recumbency. The technician positioned on the left side of the female then grasped the prepuce, cleaned the preputial orifice and directed the erect penis into the AV for copulation. During copulation, the tight feeling of the cervix was imitated manually by holding the latex liner between the AV and semen collection vessel.

### Semen evaluation

#### Initial semen assessment

Following collection, samples were transferred immediately to a 35°C water bath. Gross activity was described as the oscillatory activity of spermatozoa in an undiluted semen sample and was assessed by examining a drop of neat semen on a pre-warmed slide under a phase contrast microscope (magnification: x100, Olympus BX20, Tokyo, Japan). It was scored from 0–3; 0—no oscillatory activity; 1—slow oscillatory activity; 2—moderate oscillatory activity; 3—rapid oscillatory activity. Only ejaculates scoring a 2 or 3 qualified for inclusion in the experiments.

Samples were diluted 1:1 with pre-warmed extender and incubated in a 35°C water bath for liquefaction. Liquification status was assessed by pipetting at 5 min intervals. The average liquefaction of semen occurred 17.5 ± 0.6 min after 1:1 dilution with extender [21]. After complete liquefaction, sperm concentration was determined using a Makler Counting Chamber (Sefi-Medical Instruments, Haifa, Israel) [22].

#### Assessment of sperm motion characteristics

Motion characteristics of spermatozoa were evaluated using computer assisted sperm assessment (CASA; CEROS, Version12, Hamilton Thorne Biosciences, USA) as described previously [21]. The following CASA parameters were used; frame rate = 60 Hz; number of frames acquired = 45; minimum contrast = 55; minimum cell size (pixels) = 6; medium average path velocity cut-off = 30 μm/s; medium threshold straightness = 60%; slow average path velocity cut-off = 10 μm/s; slow straight line velocity cut-off = 5 μm/s; static average path velocity cut-off = 4 μm/s; static straight line velocity cut-off = 1 μm/s; non-motile head size (pixels) = 0.5 to 4.8; non-motile head intensity = 0.25 to 1.8; magnification = 1.87x; video frequency = 60; illumination intensity = 2300; temperature = 37°C.

Diluted, liquefied semen samples were further diluted with extender to 50 × 10^6^ spermatozoa/mL. Three microliters of semen was placed on a pre-warmed 20 μm standard count analysis chamber (Leja, Nieuw-Vennep, The Netherlands). Five randomly selected microscopic fields were scanned and approximately 500 spermatozoa counted. The total motility (TM), progressive motility (PM), average path velocity (VAP), progressive velocity (VSL) and track speed (VCL), lateral head amplitude (ALH), beat cross frequency (BCF), straightness (STR) and linearity (LIN) of spermatozoa were analysed with camel settings [21].

#### Assessment of sperm viability and acrosome integrity

Sperm viability and acrosome integrity (VIA) was evaluated as described previously [23]. Briefly, 90 μL of semen (50 x 10^6^ spermatozoa/mL) was mixed with 10 μL of 1% neutral buffered formalin at room temperature to fix and immobilize spermatozoa. Fixed spermatozoa (100 μL) were stained with 6 μL of fluorescent isothiocyanate-conjugated lectin from Arachis hypogaea (working concentration 40 μg/mL; FITC-PNA; Sigma) and incubated at 37°C for 10 min in the dark. Propidium iodide (0.5 μL; working concentration 0.6 mM; PI, Sigma) was then added and the sample incubated for another 5 min. Stained spermatozoa (20 μL) was then placed onto a glass slide and covered with a 22×40 mm coverslip. At least 200 spermatozoa per sample were examined with an Olympus BX51 epifluorescence microscope. Spermatozoa stained with both PI and FITC-PNA were classified as non-viable with damaged membranes and a non-intact acrosome. Spermatozoa stained with only PI were considered as non-viable with intact acrosomes. Unstained spermatozoa were classified as viable with intact membranes and acrosomes. Spermatozoa stained with FITC-PNA alone were classified as viable but with a non-intact acrosome.

### Liquid storage

Undiluted ejaculates were divided and diluted (1:1) with Triladyl and Optixcell. After initial semen dilution (1:1) and complete liquefaction, sperm concentration was adjusted to 100 × 10^6^ spermatozoa/mL. Semen samples were kept in a water jacket filled with 200 mL of 35°C water and cooled over 2 h, in a cold cabinet (IMV Technologies) to 4°C and stored for 24 h. Sperm motion characteristics (CASA), viability and acrosome integrity (fluorescent microscopy) were assessed at 37°C, before storage at 4°C (0 h) and 24 h.

### Cryopreservation

Whole ejaculates were diluted (1:1) with Triladyl. After complete liquefaction, sperm concentration was adjusted to 100 × 10^6^ spermatozoa/mL. Semen samples were incubated at 4°C for 3 h in a cold handling cabinet (IMV, France) for equilibration. Equilibrated semen samples were filled in 0.5 mL French straws and frozen at 5°C/min from +4 to -12°C and at 50°C/min from -12 to -140°C using programmable freezer (Minitube, Germany), and then straws were plunged into LN_2_ for storage. Samples were thawed at 37°C for 1 min. Sperm motion characteristics (CASA), viability and acrosome integrity (fluorescent microscopy) were assessed at 37°C, before equilibration and immediately after thawing.

### Management of female camels

Follicular development was synchronized to carry out natural mating (NM) or AI using the FWsynch protocol [11]. Briefly, camels received GnRH treatments on Days 0 and 10 (100 μg of GnRH, i.v., Cystorelin, Ceva Sante Animale, Libourne, France) and PGF_2α_ treatments on Days 7 and 17 (500 μg of PGF_2α_ analogue, i.m., Estrumate, Schering-Plough Animal Health, New South Wales, Australia). On Day 22, camels were scanned to confirm the presence of a dominant follicle (DF) and only camels with a DF of 11–17 mm (considered as synchronized) were used in all experiments. Camels were bred by natural mating (experiment 1) or ovulation was induced by intravenous administration of GnRH (Cystorelin, France) on Day 22.

Artificial insemination was performed in female camels in a standing position. The animals were restrained in suitably designed crates, the tail was wrapped and the rectum was emptied of faeces. The perineum was then scrubbed using a 2% iodine solution, rinsed carefully with clean water and then dried. A sterile deep intrauterine insemination pipette with inner catheter (Cat. No. 17207/1265, Minitube, Germany) was used. The external end of pipette was attached to a sterile 2–10 mL syringe (depending on volume of semen). The pipette was covered with a sterile sanitary sheath after loading the semen. Semen was deposited in the body of the uterus just cranial to cervix, by means of a manually guided pipette passed through the cervix, or at the tip of the horn ipsilateral with the ovary containing the DF, by manually guided pipette passed through the cervix and then to the tip of the horn per rectum. Early pregnancy diagnosis was performed by ultrasonography 19 ± 2 days after breeding. Female camels that were diagnosed as pregnant during this diagnosis were examined by ultrasound at 45 ± 5 days after breeding to confirm pregnancy and to record embryonic loss rates. Pregnancy was calculated by the number of camels pregnant after AI, divided by the total number of camels inseminated. Pregnancy loss was calculated by the number of camels that lost pregnancy between Day 19 ± 2 and Day 45 ± 5 of gestation divided by the number of pregnant camels on Day 19 ± 2.

### Experiment 1: Comparison of natural mating and high dose AI with fresh undiluted semen on pregnancy rate

Female camels (n = 45) were divided randomly into four groups. In group A camels (n = 12), each camel was bred by NM with one of the three fertile bulls on Day 22 (Day 0 = day of initiation of the FW synch protocol). In group B camels (n = 11), whole undiluted ejaculate (volume: 3–8 mL) from each bull was used for AI of an individual female. In group C camels (n = 10), the ejaculate from each bull was split into two portions and each portion (volume: 1.5–3.5 mL) was used for individual animal AI. In group D camels (n = 12), the ejaculate (only ejaculates with volume of ≥3.5 mL were used) from each bull was split into 1 mL doses and each dose was used for AI. Semen was deposited in the body of the uterus in all groups immediately after ovulation treatment (0 h) on Day 22. Fresh undiluted ejaculates were used within 10–15 min of collection and 0.5 mL semen sample was taken from the each ejaculate for semen analysis. Semen samples were diluted with Triladyl and TM (%), VAP (μm/s) and VIA (%) of spermatozoa was recorded at 2 h after dilution for each ejaculate used in groups B, C and D. Camels were scanned 48 h after breeding or ovulation treatment to record ovulation status.

### Experiment 2: Effect of site and timing of insemination after ovulation induction on pregnancy rate

This experiment was performed in two stages. In stage 1, fresh semen diluted with Triladyl (1:1) and containing 300 x 10^6^ motile spermatozoa [volume (mean ± SEM): 1.7 ± 0.03 mL)] was deposited in either the body of uterus (n = 17 females) or the tip of the uterine horn (n = 18 females), immediately after ovulation treatment. In stage 2, the same dose of semen was deposited in the tip of the horn at either the time of ovulation treatments (n = 19 females, 0 h), 24 (n = 20 females) or 30 h (n = 17 females) later. Semen samples from each bull were processed separately and were used for AI within 2 h after semen collection and dilution. The average TM (%), VAP (μm/s) and VIA (%) of spermatozoa in the ejaculates used in the both stages was recorded. Camels were scanned at the time of AI and 48 h after ovulation treatment to confirm ovulation status.

### Experiment 3: Effect of insemination dose and different extenders used for semen dilution on pregnancy rate

This experiment was performed in two stages. In the first stage, Triladyl diluted (1:1) and liquefied semen samples were further diluted with the same extender to 100 × 10^6^ spermatozoa/mL. An inseminate dose consisting of either 300 x 10^6^ [volume (mean ± SEM): 3.5 ± 0.04 mL, n = 20 females], 150 x 10^6^ [volume (mean ± SEM): 1.7 ± 0.02 mL, n = 23 females] or 75 x 10^6^ [volume (mean ± SEM): 0.9 ± 0.01 mL, n = 22 females] motile spermatozoa was deposited in the tip of the uterine horn at 24 h after ovulation induction treatment. The average TM (%), VAP (μm/s) and VIA (%) of spermatozoa in ejaculates used in this stage of the experiment was recorded. In the second stage, the ejaculate was divided and diluted (1:1) with either Green buffer (n = 16 females), Optixcell (n = 17 females) or Triladyl (n = 14 females) extender. An inseminate dose consisting of 150 x 10^6^ motile spermatozoa [volume (mean ± SEM): 1.7 ± 0.08 mL] from one of the treatment groups was deposited in the tip of the horn at 24 h after ovulation treatment. Semen samples from each bull were processed separately and were used for AI within 2 h after semen collection and dilution. Semen quality was assessed in all extenders at 2 h after dilution. Camels were scanned at the time of AI and 48 h after ovulation treatment to confirm ovulation status.

### Experiment 4. Effect of chilled and frozen thawed semen on pregnancy rate

In the first stage of this experiment, the ejaculate was divided and diluted in Triladyl or Optixcell extenders. Diluted semen samples were stored at 4°C for 24 h. Chilled semen (24 h stored) consisting of 300 x 10^6^ [volume (mean ± SEM): 4.0 ± 0.05 mL; Triladyl, n = 18 females; Optixcell, n = 17 females] and 500 x 10^6^ [volume (mean ± SEM): 6.7 ± 0.09 mL; Triladyl, n = 19 females; Optixcell, n = 16 females] motile spermatozoa was used for AI. Semen quality was evaluated at 0 and 24 h of liquid storage. In the second stage, frozen thawed semen consisting of 300 x 10^6^ [volume (mean ± SEM): 4.8 ± 0.08 mL, n = 23 females] and 500 x 10^6^ [volume (mean ± SEM): 7.9 ± 0.1 mL, n = 20 females] motile spermatozoa was used for AI. Semen quality was evaluated immediately after thawing. Semen was deposited in the tip of the uterine horn at 24 h after ovulation treatment in all groups. Camels were scanned at the time of AI and 48 h after ovulation treatment to confirm ovulation status.

### Statistical analysis

All statistical analyses were performed in GENSTAT (Version 17, VSN International, Hemel Hempstead, UK). Data relating to the percentage of pregnancy were statistically analysed by binomial logistic regression using a generalised linear mixed model (GLMM). Data recorded on the motion characteristics and membrane acrosome status were analysed using a linear mixed model. Extender and time of analysis were specified as a fixed effect. Ejaculates were specified as a random effect. For all models, *P <* 0.05 was considered statistically significant and if there were no significant interactions between fixed factors, the interaction was dropped from the model.

## Results

### Experiment 1: Comparison of natural mating and high dose AI with fresh undiluted semen on pregnancy rate

Pregnancy rates were similar between camels bred by NM (10/12; 83.3%) and AI with whole ejaculate (9/11; 81.8%; *P > 0*.*05*). AI with split undiluted ejaculate (6/10; 60%; *P < 0*.*05*) had a lower pregnancy rate than AI with whole ejaculate or NM. AI with 1 mL undiluted ejaculate resulted in a lower pregnancy rate than all other groups (4/12; 33.3%; *P < 0*.*05*). The average characteristics of ejaculates used for insemination in groups B, C and D are presented in Table 1. The embryonic loss in naturally mated and artificially inseminated camels was 10% (1/10) and 10.5% (2/19, data pooled from group B, group C and group D), respectively.

**Table 1:** Mean (±SEM) characteristics of ejaculates used for insemination in experiment 1.

	No. of ejaculates	Volume (mL)	Concentration (x10^6^sperm/ mL)	TM (%)	VAP (μm/s)	VIA (%)
**Group B** (AI with whole undiluted ejaculate)	11	5.1 ± 0.5	474.3 ± 27.5	86.1±1.0	113.3±3.6	69.0±1.2
**Group C** (AI with split undiluted ejaculate)	5	4.9 ± 0.4	442.9 ± 24.8	84.4±0.6	117.0±2.7	66.1±1.8
**Group D** (AI with 1mL of undiluted ejaculate)	4	4.8 ± 0.3	454.3 ± 20.2	87.6±0.8	123.6±2.5	70.9±1.6

TM, total motility; VAP, average path velocity and VIA, viable-intact acrosome.

### Experiment 2: Effect on pregnancy rate of site and timing of insemination after ovulation induction

**Fig 1:**
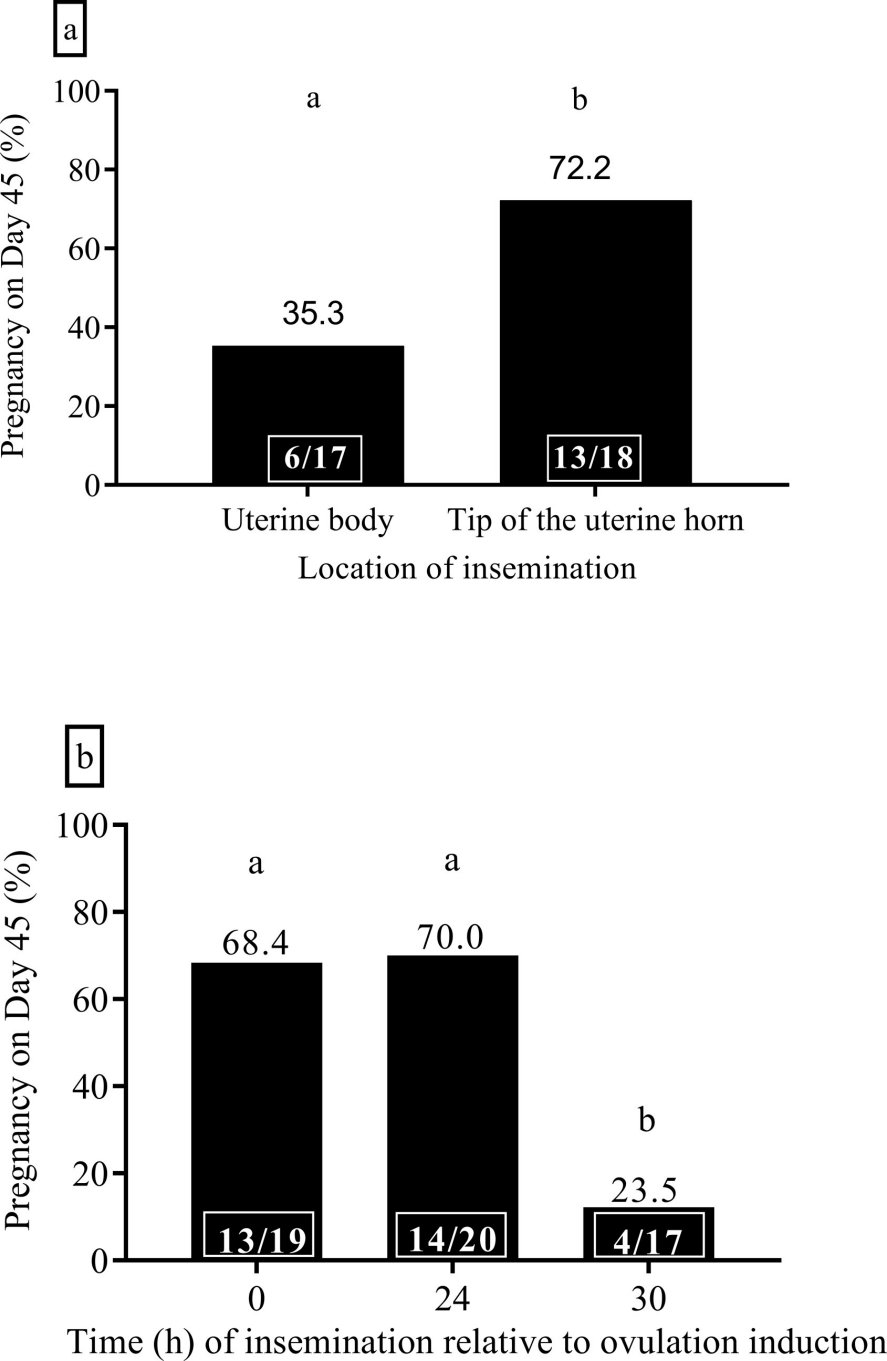
**The effect of site (a) and timing (b) of insemination on the pregnancy rate of dromedary camels using fresh diluted camel semen.** Insemination dose was 300 x 10^6^ motile spermatozoa. Bars with different superscripts differ (*P < 0*.*05*). The number in the box indicates the number of pregnant camels out of the total number of camels inseminated.

Pregnancy rate in camels inseminated immediately after ovulation induction in the body of uterus with 300 x 10^6^ motile spermatozoa was significantly lower compared to camels inseminated in the tip of the uterine horn with the same sperm numbers (35.3% *versus* 72.2%, Fig 1A, *P < 0*.*05*). The average TM, VAP and VIA of spermatozoa in the ejaculates was 86.3 ± 0.6%, 118.1 ± 2.7 μm/s and 69.5 ± 1.6%, respectively.

Insemination of camels (300 x 10^6^ motile spermatozoa) at 0 h (immediately after ovulation treatment) and 24 h after ovulation treatment resulted in a higher pregnancy rate to camels inseminated 30 h after ovulation treatment (Fig 1B, *P < 0*.*05*). The average TM, VAP and VIA of spermatozoa in the ejaculates used was 88.1 ± 1.5%, 118.5 ± 4.5 μm/s and 61.7 ± 1.8%, respectively. There was no difference in embryo loss between groups and the overall embryonic loss was 15.2% (7/46) in this experiment (data pooled from all groups).

### Experiment 3: Effect of insemination dose and different extenders used for semen dilution on pregnancy rate

**Fig 2:**
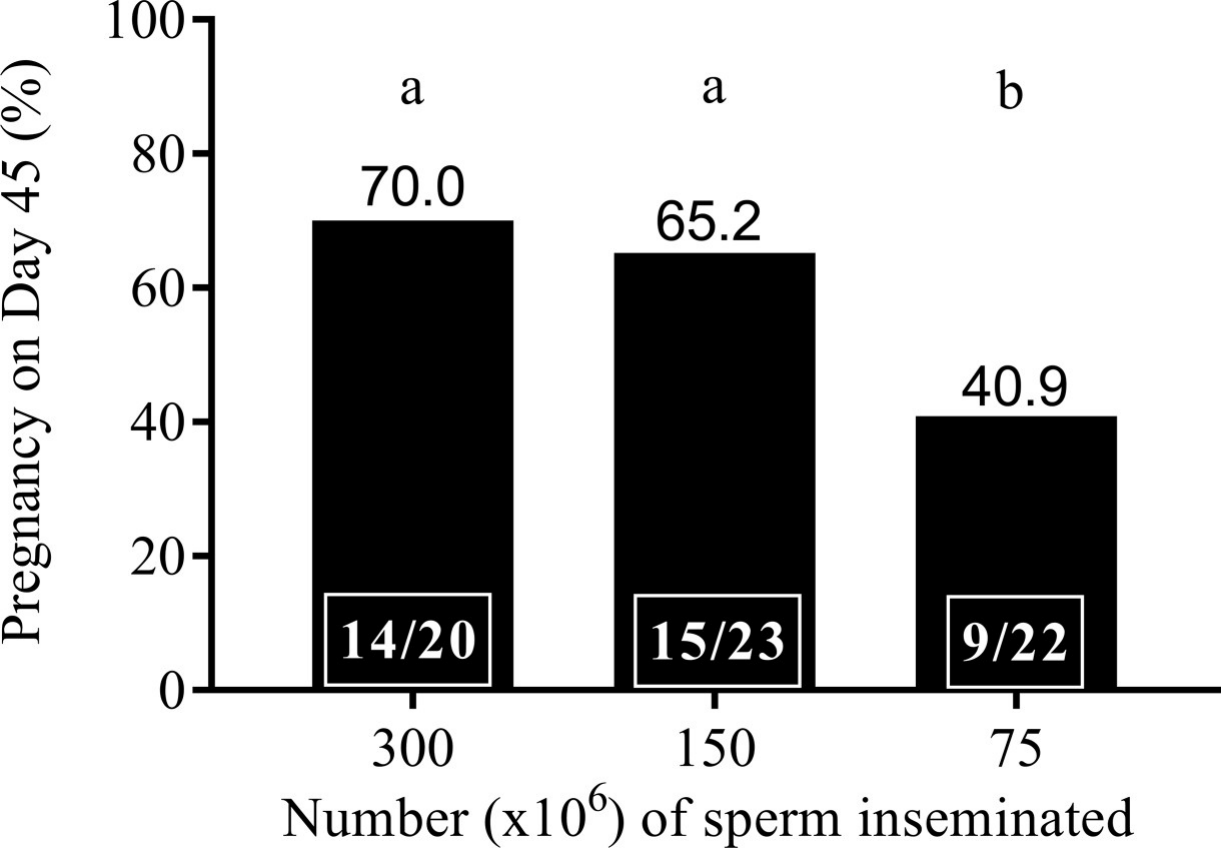
The pregnancy rate of dromedary camels following insemination with fresh diluted semen containing 300 × 10^6^, 150 × 10^6^ or 75 × 10^6^ motile spermatozoa, 24 h after ovulation induction. Bars with different superscripts differ (*P < 0*.*05*). The number in the box indicates the number of pregnant camels out of the total number of camels inseminated.

Deep horn AI with 75 x 10^6^ motile spermatozoa (40.9%) resulted in a significantly lower pregnancy rate compared with deep horn AI of 150 x 10^6^ (65.2%) and 300 x 10^6^ (70%) motile spermatozoa (Fig 2, *P < 0*.*05)*. The average TM, PM and VIA of spermatozoa in ejaculates used in this stage of the experiment was 86.5 ± 0.9%, 100.1 ± 2.8 μm/s and 61.6 ± 1.3%, respectively.

In the second stage of the experiment, the average TM, VAP and VIA of spermatozoa did not differ significantly (*P > 0*.*05*) among Green buffer (84.7 ± 1.0%, 95.5 ± 3.8 μm/s and 62.8 ± 1.0%, respectively), Optixcell (87.0 ± 1.3%, 104.3 ± 8.3 μm/s and 63.0 ± 1.8%, respectively) and Triladyl (84.1 ± 0.9%, 100.5 ± 3.8 μm/s and 66.2 ± 1.6%, respectively). The pregnancy rate was similar among camels inseminated with Green buffer or Optixcell or Triladyl diluted semen (68.8% versus 64.7% versus 64.3%, *P > 0*.*05*). Overall embryonic loss was 17.2% (11/64) in this experiment (data pooled from all groups, as there was no difference between groups).

### Experiment 4. Effect of chilled and frozen thawed semen on pregnancy rate

The TM, VAP and percent viable, acrosome intact spermatozoa diluted in Triladyl and Green buffer was similar at 0 (*P > 0*.*05*, Table 2). At 24 h, Triladyl recorded significantly higher TM and VAP compared to Green buffer (*P < 0*.*05*, Table 2).

**Table 2:** Mean (±SEM) total motility, average path velocity and percentage of viable intact acrosomes of spermatozoa in ejaculates (n = 26) diluted in two different extenders and evaluated 0 and 24 h of liquid storage at 4°C in experiment 4.

Extenders	TM (%)		VAP (μm/s)		VIA (%)	
	0 h	24 h	0 h	24 h	0 h	24 h
**Optixcell**	86.4±1.0	72.8±1.4^a^	101.9±3.3	81.5±3.3^a^	65±1.3	53.8±1.7
**Triladyl**	88.5±0.7	76.8±1.2^b^	106.4±2.8	96.3±2.6^b^	64.8±1.3	57.9±1.2

TM, total motility; VAP, average path velocity and VIA, viable-intact acrosome. Values within a column with different superscripts differ (*P < 0*.*05*).

Pregnancy rates obtained following insemination at the tip of the uterine horn of 300 x 10^6^ or 500 x 10^6^ 24 h chilled spermatozoa in Triladyl or Optixcell were similar (Triladyl; 11.1% versus 21.1% and Optixcell; 5.9% versus 12.5%) for 300 x 10^6^ and 500 x 10^6^ groups, respectively; P > 0.05).

The average TM, PM and VIA of spermatozoa before freezing (fresh semen) were 86.2 ± 0.6%, 112.7 ± 2.2 μm/s and 68.9 ± 1.1%, respectively. The average TM, PM and VIA of spermatozoa in frozen thawed semen were 63.4 ± 1.0%, 74.3 ± 1.7 μm/s and 44.0 ± 1.1%, respectively. No pregnancies were obtained following insemination at the tip of the uterine horns with either 300 x 10^6^ or 500 x 10^6^ motile frozen-thawed spermatozoa.

## Discussion

The present study is the first to comprehensively investigate the effect of site of insemination, time of insemination relative to ovulation induction, inseminate dose, different extenders used for semen dilution and type of semen on the success of AI of the dromedary camel. Overall, results showed that pregnancy rates of >50% could be achieved by inseminating fresh semen consisting of 150 x 10^6^ motile spermatozoa in the tip of the uterine horn. Chilled semen resulted in poor fertility (<50%) and no pregnancies were achieved with frozen-thawed semen. Furthermore, this study demonstrated that the site of insemination and sperm dose used for insemination influenced pregnancy rates.

The finding that insemination of whole undiluted ejaculates into the body of the uterus resulted in similar pregnancy rates to timed natural service (81.8% *versus* 83.0%, respectively) demonstrated that the procedure of AI itself does not compromise fertility in camels. The pregnancy rate reported in the present study was higher compared to the pregnancy rate reported with insemination of whole undiluted semen in previous studies (40–46.1%, [4, 10]). However, insemination with a split ejaculate or 1 mL of undiluted ejaculate into the body of the uterus caused a significant drop in the pregnancy rate (split ejaculate: 60% and 1 mL undiluted ejaculate: 33.3%). These findings indicate that the success of AI is highly sensitive to inseminate volume or more likely, the number of spermatozoa deposited in the reproductive tract.

Insemination of 300 x 10^6^ motile spermatozoa in the tip of horn resulted in a significantly higher pregnancy rate than when deposited lower down the tract in the body of the uterus. However, a previous study reported no difference in pregnancy rates after AI of 150 x 10^6^ motile spermatozoa into the uterine horn compared with the uterine body, but a significantly higher pregnancy rate after AI into the tip of the horn compared to the body of uterus when only 80 x 10^6^ motile spermatozoa were used [15]. The lower pregnancy rate achieved following insemination in the body of the uterus could be due to loss of spermatozoa via backflow of semen through the cervix. In any event, the aforementioned observations indicate that insemination into the tip of the horn is ideal in this species although the procedure requires a skilled AI technician.

The timing of insemination in relation to ovulation, dose of spermatozoa used for AI and extenders used for fresh semen dilution are also important aspects of insemination in camelids. A study from our centre which examined the interval from ovulation treatment to ovulation by ultrasonographic scanning every 8 h following ovulation treatment showed that ovulation occurs at 32.2 ± 0.4 h (mean ± S.E.M) after ovulation treatment (i.e., GnRH or hCG) in dromedary camels [24]. Insemination at 0 and 24 h after ovulation induction resulted in similar pregnancy rates in this study. This is in contrast to previous studies reporting better conception rates with insemination at 24 h after ovulation treatment than at the time of treatment (56% *versus* 36%, [17], 58.3% *versus* 48% [18]). Some groups have carried out insemination at 30 h [20], 48 h [10] and two inseminations at 24 and 48 h after ovulation treatment in dromedary camels [4]. A study in alpacas showed more desirable results with insemination at 35–45 h than immediately or 24 h after ovulation treatment [25]. In the present study, insemination at 30 h after ovulation treatment resulted in poor pregnancy rates (23.5%, Fig 1B) and all camels had ovulated before carrying out insemination. The reason for the different results of the present study and those of other authors is difficult to isolate, but may be due to differences in semen handling and/or technique of insemination. The present study has demonstrated that AI should be done at the time ovulation induction or 24 h later.

In the present study, pregnancy rate was affected by the number of spermatozoa inseminated. The pregnancy rate was similar between camels inseminated into the tip of the horn with 300 x 10^6^ and 150 x 10^6^ spermatozoa (70 and 65.2%, respectively) but reduced when the insemination dose was dropped to 75 x 10^6^ motile spermatozoa (40.9%). In a previous study, insemination of 150 x 10^6^ and 80 x 10^6^ motile spermatozoa in the tip of the horn resulted in similar pregnancy rates (43 and 40%, respectively [15]) and insemination of 300 x 10^6^ motile spermatozoa in the body of uterus resulted in higher pregnancy rate than 150 x10^6^ motile spermatozoa deposited in this same location (41.6 and 27.2%, respectively) [8]. The higher pregnancy rate reported in the present study compared to previous studies could be attributed to the quality of motile spermatozoa and site of insemination. Among different extenders used for semen dilution, the pregnancy rate was not affected in the present study. Similarly, [8] reported similar pregnancy rates with semen diluted in Green Buffer and INRA 96. These observations indicate that egg yolk based Green buffer and Triladyl and chemically defined extenders Optixcell and INRA 96 can be used to dilute fresh semen without affecting fertility.

The pregnancy rate achieved with insemination of 150 x 10^6^ motile spermatozoa into the tip of the horn is comparable with pregnancy rate achieved with timed natural service programmes [11]. This shows that the minimum effective insemination dose is 150 x 10^6^ motile spermatozoa in dromedary camels. There is limited data on early pregnancy loss in camels that conceived following insemination. Overall in the present study, 15.2–17.2% early pregnancy loss was recorded. The early embryonic losses recorded in the present study were comparable with early pregnancy losses recorded in timed natural service programmes [11]. This indicates that semen collection, handling and insemination method is not altering the uterine environment.

Use of chilled or frozen-thawed semen helps enable adoption of AI as a technique of breeding in field conditions and also facilitates semen transport domestically and internationally. Several studies have demonstrated liquid storage of dromedary camel semen at 4°C [8, 10, 26, 27] and cryopreservation of semen [4, 19, 20]. However, there is a dearth of literature regarding insemination protocols using chilled and frozen-thawed semen. In our previous study, among several extenders tested for liquid storage of semen at 4°C, Optixcell, Green buffer and Triladyl were found to be successful in preserving semen up to 48 h [28]. In the present study, although liquid stored (24 h) semen was good when evaluated in vitro, poor pregnancy rates (5.9–21.1%) were recorded following insemination. Similarly, previous studies reported poor pregnancy rates following insemination of chilled and stored semen in dromedary camels [4, 8], alpacas [29], and llamas [30]. Our results and above observations indicate that liquid storage is affecting the fertilizing capacity of spermatozoa, but the underlying cause is unknown especially when considering in vitro semen quality appears to be high.

In the present study, although post-thaw semen showed good quality parameters of 63.4 ± 1.0% TM, 74.3 ± 1.7 μm/s VAP and 44.0 ± 1.1% VIA spermatozoa, no pregnancies were established following insemination. However, a recent study has reported a 62% pregnancy rate (excluding 28.5% early embryonic death and 9.5% mid-gestation abortions), following frozen-thawed insemination at 30 h after ovulation inducing treatment [20]. Details on sperm dose and site of insemination were not provided for this study [20]. At our centre, no pregnancies were obtained by inseminating frozen thawed semen at 28–30 h after ovulation treatment (unpublished observations, S. Al-Bulushi). Given the results reported herein and those of other groups, the results of Akbar et al. (2018) should be viewed with caution. Although motion and viability and acrosome integrity parameters have been evaluated in chilled and frozen-thawed semen, other in vitro parameters such as sperm capacitation status, DNA and mitochondrial membrane potential were not assessed. These parameters might have been more useful in predicting fertility of semen.

In conclusion, deep horn insemination with fresh semen consisting of 150 x 10^6^ motile spermatozoa recorded >60% pregnancy rates. This demonstrates that fresh AI can be undertaken at the time ovulation or 24 h later. Green buffer, Optixcell and Triladyl extenders can be used for fresh semen dilution and insemination within 2 h after collection and dilution. Use of chilled semen for AI recorded poor fertility rates and with frozen-thawed semen no pregnancies were recorded.
